# Serum Angiopoietin-2 Predicts the Occurrence and Recurrence of Hepatocellular Carcinoma after Direct-Acting Antiviral Therapy for Hepatitis C

**DOI:** 10.3390/v15010181

**Published:** 2023-01-07

**Authors:** Naoki Kawagishi, Goki Suda, Yoshiya Yamamoto, Masaru Baba, Ken Furuya, Osamu Maehara, Shunsuke Ohnishi, Sonoe Yoshida, Qingjie Fu, Zijian Yang, Shunichi Hosoda, Yoshimasa Tokuchi, Takashi Kitagataya, Masatsugu Ohara, Kazuharu Suzuki, Masato Nakai, Takuya Sho, Mitsuteru Natsuizaka, Koji Ogawa, Naoya Sakamoto

**Affiliations:** 1 Department of Gastroenterology and Hepatology, Graduate School of Medicine, Hokkaido University, Sapporo 0608638, Japan; 2 Department of Gastroenterology, Hakodate Municipal Hospital, Sapporo 0608638, Japan; 3 Department of Gastroenterology and Hepatology, Japan Community Health Care Organization (JCHO) Hokkaido Hospital, Sapporo 0608638, Japan; 4 Laboratory of Molecular and Cellular Medicine, Faculty of Pharmaceutical Sciences, Hokkaido University, Sapporo 0608638, Japan

**Keywords:** hepatitis C virus, direct-acting antiviral, angiopoietin-2, hepatocellular carcinoma, recurrence

## Abstract

Progressive liver fibrosis after anti-HCV treatment is a risk factor for HCC. Angiopoietin-2 (Ang2) is associated with non-regression of liver fibrosis after direct-acting antiviral (DAA). This study evaluated the predictive value of serum Ang2 levels for HCC occurrence or recurrence after DAA administration. In this retrospective study, 310 HCV-infected patients treated with DAAs in 2014–2020 were screened and evaluated for HCC occurrence or recurrence every three–six months. Multivariate Cox regression analysis revealed that age ≥ 75 years (HR: 2.92, 95% CI: 1.34–6.33; *p* = 0.007) and baseline Ang2 level ≥ 464 pg/mL (HR: 2.75, 95% CI: 1.18–6.37; *p* = 0.019) were significantly associated with HCC occurrence after DAA therapy. A high or low risk of HCC after DAA therapy could be distinguished by the combination of age and baseline Ang2 level. The cumulative incidences of de-novo HCC at two and four years were 0.8% and 3.8% in the low-risk group and 22.6% and 27.1% in the high-risk group, respectively. Baseline Ang2 level ≥ 402 pg/mL was significantly associated with HCC recurrence in patients who achieved sustained virological response with DAAs (HR: 3.68). In conclusion, serum Ang2 levels can predict HCC occurrence and recurrence after successful HCV eradication by DAAs.

## 1. Introduction

Direct-acting antivirals (DAAs) have strongly impacted hepatitis C virus (HCV) treatment. Clinical trials and real-world data have proven that most patients can achieve sustained virological response (SVR) by DAAs, including HCV-infected patients with complications associated with a high risk of hepatocellular carcinoma (HCC) [1,2,3,4,5,6,7,8,9,10,11,12]. Although recent studies have revealed that successful HCV eradication by DAAs could prevent the occurrence of HCC similar to the IFN treatment [13], HCC occurrence and recurrence, even after successful HCV eradication, remain a clinically important issue. Given that numerous patients with a high risk of HCC could achieve SVR by DAAs, precise and simple methods for predicting HCC occurrence after successful HCV eradication by DAAs and detailed mechanistic analyses are required.

Even after successful HCV eradication, progressive liver fibrosis has been identified as a risk factor for HCC occurrence after SVR [14]. The majority of patients who achieve SVR with anti-HCV therapy exhibit restoration of the hepatic functional reserve and regression of the liver fibrosis [14,15]. However, patients who do not exhibit regression of liver fibrosis [14,15,16,17] are at high risk for HCC occurrence after successful HCV eradication [14]. Recent reports suggest that angiopoietin-2 (Ang2) can predict non-regression of liver fibrosis after successful HCV eradication [16,18].

Angiopoietin-1 (Ang1) is expressed mainly in mesenchymal cells and has the potential to act as an agonist of Tie2-mediated signaling, which is associated with vessel stabilization and endothelial barrier function [19,20]. However, Ang2 is mainly expressed in endothelial cells [21] and has an antagonistic effect on Ang1-Tie2 signaling; thus, high expression levels of Ang2 could cause vessel destabilization, decreased endothelial barrier function, and inflammation.

Taken together, non-regression of liver fibrosis after HCV eradication could be predicted by Ang2 and might be a risk factor for HCC.

In this study, we evaluated the hypothesis that serum Ang2 levels might predict HCC occurrence and recurrence after successful HCV eradication by DAAs.

## 2. Materials and Methods

### 2.1. Study Design

Consecutive HCV-infected patients who were treated with DAAs between October 2014 and January 2020 at Hokkaido University Hospital, Japan Community Health Care Organization Hokkaido Hospital, and Hakodate Municipal Hospital were screened. Patients were included if they were treated with an interferon-free DAA regimen, had preserved serum for the analyses of Ang2, were followed for >1 year from the start date of DAA treatment, had proper clinical information, were screened for HCC occurrence or recurrence every three–six months after DAA completion by ultrasonography, computed tomography (CT), or magnetic resonance imaging (MRI), and achieved SVR by DAAs. Patients were excluded if they did not have preserved serum for analyses of Ang2 at baseline, did not achieve SVR, were not screened for HCC occurrence or recurrence properly, were diagnosed with HCC within 180 days from the start date of DAA treatment, had a history of non-curative treatment for HCC, and did not have proper clinical information. In this study, patients with HCV infection who were treated with DAAs visited the attending physician every three–six months after completion of DAA therapy. Laboratory data were evaluated, and imaging tests were performed using echocardiography, CT, or MRI to detect HCC occurrence and recurrence. In this study, referring to the previous studies [22,23,24], liver cirrhosis was diagnosed by laboratory findings, liver histology, radiologic findings, and/or liver stiffness data using FibroScan (Echosens, Paris, France).

Factors, including clinical factors and serum biomarkers, which were associated with HCC occurrence and recurrence after successful HCV eradication by DAAs, were evaluated. Using a method similar to that from previous reports, serum Ang2 levels at baseline (*n* = 310) were detected by an enzyme-linked immunosorbent assay (R&D Systems, Minneapolis, MN, USA) [16,18,25,26]. Further, serum Ang2 levels were evaluated at the end of the treatment in patients who had preserved serum samples (*n* = 284). 

This study was conducted in accordance with the principles embodied in the Declaration of Helsinki and was approved by the ethical committees of Hokkaido University Hospital and Hakodate Municipal Hospital (UMIN000031091). All enrolled patients provided written informed consent to participate in this study or did not decline to participate. The ethics committee specifically approved the non-decline of being included in the study in lieu of written informed consent for some patients.

### 2.2. Statistical Analyses

Univariate Cox regression analysis was conducted on clinical factors and laboratory data, including Ang2 values. Multivariate Cox regression analysis was performed on factors significantly associated with HCC occurrence and recurrence after DAA therapy in the univariate analysis (*p* < 0.05). The optimal cut-off values for the factors utilized in the univariate and multivariate analyses were determined using the Youden index for the receiver-operating characteristic (ROC) curve. The cumulative incidences of HCC occurrence and recurrence were evaluated using the Kaplan–Meier method and analyzed using the log-rank test.

Statistical analyses were performed using SPSS version 24.0 (IBM Japan, Tokyo, Japan) and Prism 7.03 (GraphPad Software, Inc., La Jolla, CA, USA). A *p*-value of < 0.05 was considered statistically significant.

## 3. Results

### 3.1. Baseline Patient Characteristics

Table 1 shows the characteristics of patients with or without a history of HCC. Among 310 patients included in this study, 256 patients without a history of HCC who achieved SVR by DAAs were evaluated for Ang2 at baseline and were followed for >1 year from the initiation of DAA therapy. Additionally, 54 patients with a history of curative treatment for HCC, who achieved SVR by DAAs, were evaluated for Ang2 at baseline (Table 1). A total of 232 (71%) and 76 (28%) patients had HCV genotypes 1 and 2. Furthermore, 90, 99, 70, 13, 12, and 26 patients were treated with daclatasvir plus asunaprevir, sofosbuvir plus ledipasvir, sofosbuvir plus ribavirin, ombitasvir plus paritaprevir, grazoprevir plus elbasvir, and glecaprevir plus pibrentasvir, respectively. The median observational period was 50.6 months (range, 12.1–86 months) in patients who did not have HCC from the start date of DAA treatment (Table S1).

### 3.2. Baseline Factors Associated with HCC Occurrence after Successful HCV Eradication by DAAs

**Figure 1:**
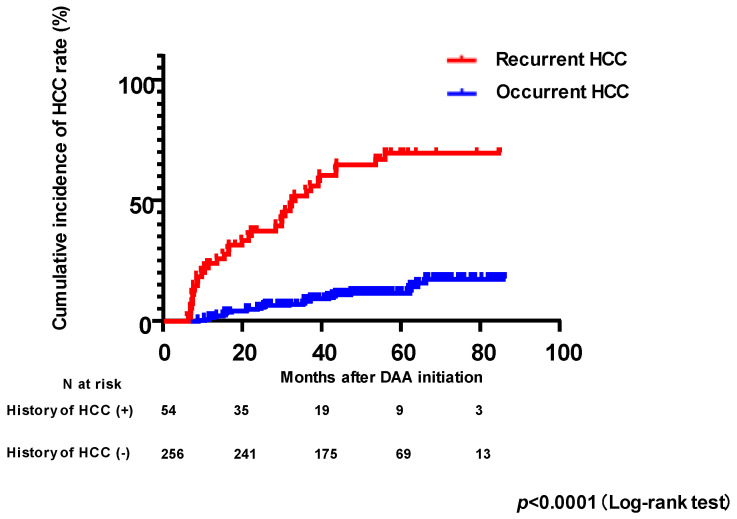
Cumulative incidences of HCC occurrence and recurrence following the initiation of DAA therapy. HCC, hepatocellular carcinoma; DAAs, direct-acting antivirals.

Of the 256 patients without a history of HCC, 31 had de novo HCC (Table 1). The cumulative incidences of HCC occurrence at two and four years were 5.1% and 11.9%, respectively (Figure 1). The univariate Cox regression analysis revealed that age ≥75 years, M2BPGi ≥ 4.23, AFP ≥ 7.7 ng/mL, Ang2 ≥ 464 pg/mL, and diabetes were significantly associated with HCC occurrence after successful HCV eradication by DAAs (Table 2). A subsequent multivariate Cox regression analysis was conducted, which included significant factors associated with HCC occurrence after HCV eradication by DAAs from the univariate analysis. The results indicated that age ≥75 years (hazard ratio (HR): 2.92, 95% confidence interval (CI): 1.34–6.33; *p* = 0.007, C-index:0.553) and baseline Ang2 level (HR: 2.75, 95% CI: 1.18–6.37; *p* = 0.019) were significantly associated with HCC occurrence after successful HCV eradication by DAAs.

### 3.3. Risk of HCC Occurrence after Successful HCV Eradication by DAAs According to Serum Ang2 Levels and Age

**Figure 2:**
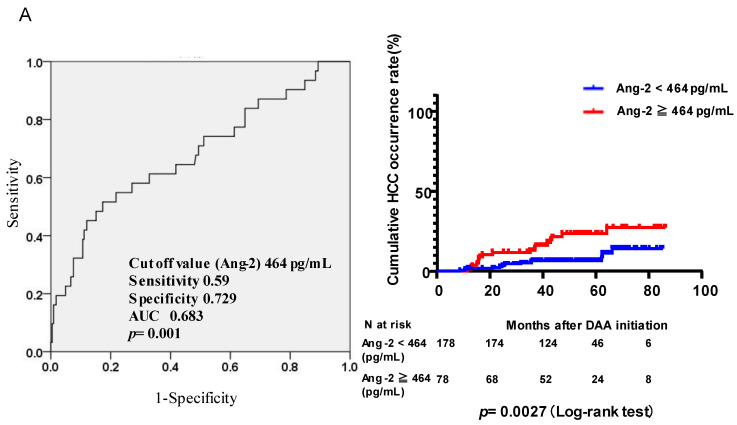

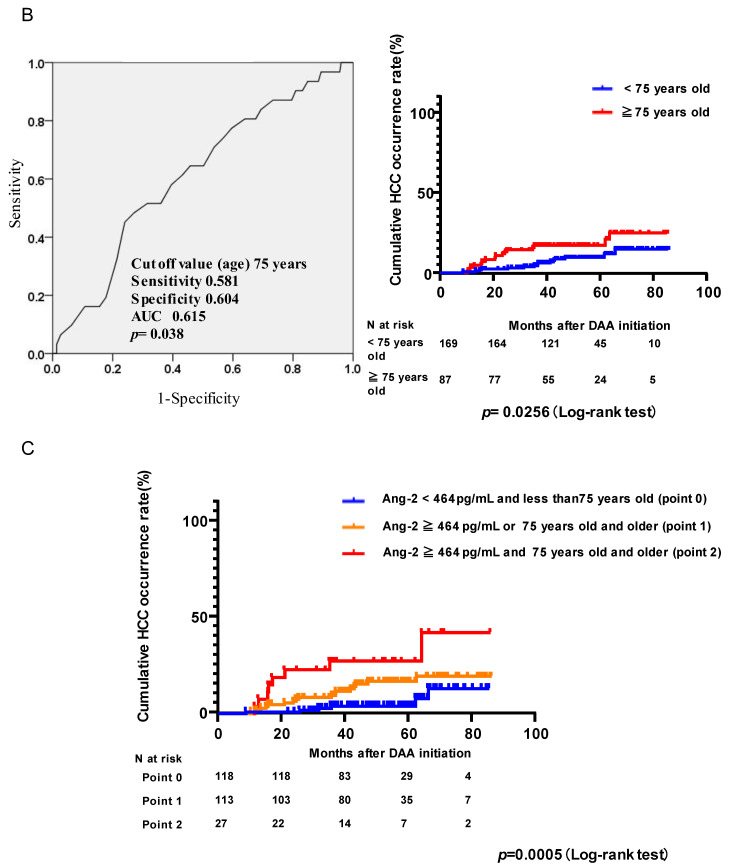
ROC curve analysis to determine the optimal cut-off values for predicting HCC occurrence and cumulative HCC occurrence according to (**A**) the serum Ang2 value and (**B**) age. (**A**) The optimal cut-off value for baseline Ang2 was set at 464 pg/mL (sensitivity, 0.59; specificity, 0.729; AUC, 0.683; *p* = 0.001). Comparison of the cumulative HCC occurrence rate (%) between patients with baseline serum Ang2 levels ≥ 464 pg/mL and <464 pg/mL. (**B**) The optimal cut-off value for baseline age was set at 75 years (sensitivity, 0.581; specificity, 0.604; AUC, 0.615; *p* = 0.038). Comparison of the cumulative HCC occurrence rate (%) between patients with baseline age ≥75 years and <75 years. (**C**) One point was assigned for baseline age ≥75 years or Ang2 level ≥ 464 pg/mL. Individuals were classified into three groups according to the total points (0, 1, and 2) to analyze the risk of HCC after successful HCV eradication. The cumulative HCC occurrence rate (%) after DAA treatment was analyzed. ROC, receiver operating characteristic; HCC, hepatocellular carcinoma; SVR, sustained virological response; DAAs, direct-acting antivirals.

We defined the optimal cut-off values for baseline age and serum Ang2 levels by receiver-operating characteristic (ROC) analyses for predicting HCC occurrence after successful HCV eradication by DAAs. The optimal cut-off value for baseline Ang2 was set at 464 pg/mL (sensitivity, 0.59; specificity, 0.729; AUC, 0.683; *p* = 0.001) (Figure 2A). A comparison of cumulative incidences of HCC occurrence (%) revealed that patients with baseline serum Ang2 level < 464 pg/mL had a significantly lower risk of HCC incidence than those with baseline serum Ang2 level ≥ 464 pg/mL (*p* = 0.0027) (Figure 2A). Estimated cumulative incidences of de novo HCC at two and four years after the initiation of DAA therapy were 11.7% and 23.5% in the Ang2 ≥ 464 pg/mL group and 3.4% and 6.6% in the Ang2 < 464 pg/mL group, respectively.

The optimal cut-off value for age was set at 75 years (sensitivity, 0.581; specificity, 0.604; AUC, 0.615; *p* = 0.038) (Figure 2B). A comparison of cumulative incidences of HCC occurrence (%) revealed that patients with a baseline age of <75 years had a lower incidence of HCC than patients with a baseline age of ≥75 years (*p* = 0.026) (Figure 2B). The cumulative incidences of HCC occurrence at two and four years were 11.7% and 16.8% in the ≥75 years old group and 2.4% and 9.4% in the <75 years group, respectively.

Subsequently, we evaluated the risk of HCC according to the combination of baseline age and Ang2 levels. We set 1 point for baseline age ≥75 years or Ang2 level ≥464 pg/mL. We classified the three groups according to the total points (0, 1, or 2) and analyzed the risk of HCC occurrence after successful HCV eradication. As shown in Figure 2C, the risk of HCC occurrence was significantly associated with the total points (0 points vs. 1 point, HR: 2.8827, 95% CI: 1.2728–6.5274, *p* = 0.0192; 1 point vs. 2 points, HR: 2.3234, 95% CI: 0.815–6.6225, *p* = 0.0425; 0 points vs. 2 points, HR: 6.4809, 95% CI: 1.61–26.0824, *p* < 0.0001) (Figure 2C). The cumulative incidences of de novo HCC at two and four years after the initiation of DAA therapy were 0.8% and 3.8% for 0 points, 5.5% and 16.4% for 1 point, and 22.6% and 27.1% for 3 points (*p* = 0.0005).

### 3.4. Factors at the End of Treatment Associated with HCC Occurrence after Successful HCV Eradication by DAAs

We analyzed the factors at the end of treatment that were significantly associated with HCC occurrence. The univariate Cox regression analysis showed that age ≥75 years, FIB-4 index ≥ 3.67, M2BPGi ≥ 1.89, AFP ≥ 4.6 ng/mL, Ang2 ≥ 402 pg/mL, and diabetes were significantly associated with HCC occurrence after successful HCV eradication by DAAs (Table 3). The multivariate Cox regression analysis revealed that Ang2 ≥ 402 pg/mL at the end of treatment alone was significantly associated with HCC occurrence after SVR by DAAs (HR: 3.68, 95% CI: 1.37–9.9; *p* < 0.01, C-index:0.55).

### 3.5. Factors Associated with HCC Recurrence after Successful HCV Eradication by DAAs

Finally, we analyzed factors associated with HCC recurrence after successful HCV eradication by DAAs in patients who had a history of HCC and were treated with curative therapy for HCC. Of the 54 patients with a history of HCC, 19 had recurrent HCC. The cumulative incidences of HCC recurrence at two and four years were 37.5% and 65.1%, respectively (Figure 1).

The univariate Cox regression analysis showed that baseline AST ≥ 45 IU/L, ALT ≥ 37 IU/L, and Ang2 levels ≥ 446 pg/mL were significantly associated with HCC recurrence after successful HCV eradication by DAAs (Table 4). The multivariate Cox regression analysis of significant factors identified in the univariate analysis revealed that a baseline Ang2 level of ≥ 446 pg/mL was significantly associated with HCC recurrence after successful HCV eradication by DAAs (HR: 2.659, 95% CI: 1.209–5.847; *p* < 0.015).

**Figure 3:**
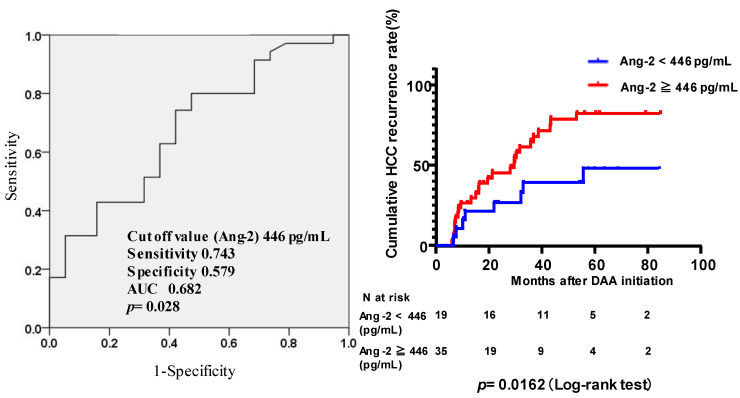
ROC curve analysis to determine the optimal cut-off values of Ang2 for predicting HCC recurrence and cumulative incidence of HCC recurrence following the initiation of DAA therapy. ROC curve analysis was used to determine the best cut-off values for predicting HCC recurrence and cumulative incidence of HCC recurrence according to the serum Ang2 value. The optimal cut-off value for baseline Ang2 was set at 446 pg/mL (sensitivity 0.743, specificity 0.579, AUC 0.682; *p* = 0.028). Comparison of the cumulative incidence of HCC recurrence (%) between patients with baseline serum Ang2 levels ≥ 446 pg/mL and <446 pg/mL.

We defined the optimal cut-off values for baseline Ang2 levels by conducting an ROC analysis for predicting HCC recurrence after successful HCV eradication by DAAs. The optimal cut-off value for baseline Ang2 was 446 pg/mL (sensitivity, 0.743; specificity, 0.579; AUC, 0.682; *p* = 0.028) (Figure 3). The cumulative incidences of HCC recurrence at two and four years were 45.2% and 78.5% in the Ang2 ≥ 446 pg/mL group and 26.3% and 38.6% in the Ang2 < 446 pg/mL group, respectively.

## 4. Discussion

We found that high baseline Ang2 levels and age were significantly associated with HCC occurrence after successful HCV eradication. Moreover, by combining age (baseline age ≥75 years or not) and baseline Ang2 levels (≥464 pg/mL or not), we could effectively distinguish individuals with high or low risk of HCC after successful HCV eradication by DAAs. Ang2 levels at the end of treatment could predict HCC occurrence. Additionally, baseline Ang2 was significantly associated with HCC recurrence in HCV-infected patients with a history of curative treatment for HCC and achieved SVR by DAAs.

DAAs have revolutionized the efficacy and safety of HCV treatment. However, even after successful HCV eradication by DAAs, HCC occurrence and recurrence are observed occasionally. Reports of HCC after successful HCV eradication by DAAs are increasing with the number of patients who achieved SVR by DAAs. Additionally, DAAs could be applied to patients with advanced liver fibrosis and elderly patients who are at high risk for HCC. In Japan, the average age of HCV-infected patients is higher than in other countries [27]. Thus, a biomarker that is easy to obtain without special equipment is required. Herein, we revealed that simple factors, such as baseline or end-of-treatment serum Ang2 levels, could predict the risk of HCC after successful HCV eradication by DAAs.

Several studies have reported the factors associated with HCC occurrence or recurrence after successful HCV eradication by DAAs, including AFP [28,29,30,31], FIB-4 index [31], Wisteria floribunda agglutinin-positive M2BPGi [28], and liver-stiffness measurement [32]. Liver-stiffness measurement requires specific equipment, whereas blood tests are easy to conduct in general practice. In this study, each potential blood biomarker (i.e., M2BPGi, AFP, and Ang2) was significantly associated with HCC occurrence in the univariate analysis; however, Ang2 alone was significantly associated with HCC occurrence and recurrence in the multivariate analysis. 

Ang1–Tie2 signaling maintains vessel integrity and endothelial barrier function [19,20]. Further, Ang2 acts as an antagonist of Ang1–Tie2 signaling, resulting in leakage of vessels and inflammation. The cytokines VEGF and TGF, hypoxia, and portal hypertension caused by liver cirrhosis can induce Ang2 [21,33,34]. High expression levels of Ang2 in liver tissues are associated with high serum Ang2 levels [34]. Thus, serum Ang2 level is a candidate biomarker for the presence of liver diseases such as nonalcoholic steatohepatitis (NASH) [35] and mortality in decompensated LC with kidney dysfunction [36].

Recently, it was reported that increased serum Ang2 levels after successful HCV eradication could predict non-regression of liver fibrosis [18]. This study found that increased serum Ang2 levels at baseline and after successful HCV eradication by DAAs were significantly associated with HCC occurrence. The progression of liver fibrosis after HCV eradication has been identified as a risk factor for the HCC [14], which was consistent with the results of this study. Moreover, high Ang2 expression in the liver tissue is a risk factor for HCC [34]. Thus, consistent with previous reports, serum Ang2 levels might predict HCC occurrence after successful HCV eradication by DAAs. 

Additionally, Ang2 has become a therapeutic target for various diseases [21]. Anti-Ang2 and anti-VEGF antibodies are effective against diabetic macular edema [21,37] and have been utilized in clinical practice. Recently, the effectiveness of the inhibition of Ang2 and VEGF in diabetic macular edema has been reported [37]. Similarly, Ang2 is a potential novel therapeutic target for liver disease. Anti-Ang2 and anti-VEGF antibodies combined with immune checkpoint inhibitors have shown beneficial effects in unresectable HCC [38]. Moreover, increased Ang2 levels were observed in patients with NASH and advanced liver fibrosis, and the inhibition of Ang2 by antibodies or small molecules could restore liver fibrosis in mouse models [35,39]. Taken together, anti-Ang2 therapy is an attractive method for preventing liver fibrosis and HCC occurrence after successful HCV eradication. Further analyses are required to confirm this hypothesis.

The present study had several limitations. First, this was a retrospective study with a small sample size and a limited observational period. In addition, due to the retrospective nature, several clinical data, including T-bilirubin, liver stiffness, and Ang-2 in HCC recurrence patients at EOT, were lacking. Therefore, a prospective study with a larger sample size is required to validate our results.

## 5. Conclusions

Serum Ang2 levels could predict HCC occurrence and recurrence after successful HCV eradication using DAAs.

## Figures and Tables

**Table 1:** Baseline characteristics of patients without or with a history of HCC.

	No History of HCC	History of HCC
Number	256	54
Age (years) ^a^	70 (22–86)	73 (50–86)
Sex (male/female)	102/154	29/25
HCV genotype (1/2/unknown)	183/71/2	49/5/0
Platelet count (10^4^/μL) ^a^	13.3 (2.2–37.3)	11.9 (1.9–38.8)
Albumin (g/dL) ^a^	4.0 (2.5–4.9)	3.7 (2.6–4.4)
AST (IU/L) ^a^	47 (16–342)	47.5 (15–176)
ALT (IU/L) ^a^	42 (6–379)	37 (14–128)
FIB-4 index ^a^	3.99 (0.54–23.55)	5.87 (1.36–39.35)
Liver cirrhosis (LC)/non-LC	123/133	36/18
M2BPGi ^a^	2.69 (0.28–17.76)	5.31 (0.67–20.01)
AFP (ng/mL) ^a^	5.4 (1.1–250.8)	8.6 (2.2–83.9)
Angiopoietin-2 (pg/mL) ^a^	367.9 (131.9–1489)	483.8 (178.2–1598.6)
Diabetes, *n* (%)	50 (19.5%)	14 (25.9%)
Duration from the start date of DAA treatment (months) ^a,b^	49.3 (12.3–86)	57.4 (12.1–85.1)
History of HCC, *n* (%)	0 (0%)	54 (100%)
Previous HCC characteristics		
HCC stage (1/2/3/4)	-	30/23/1/0
Treatment (RFA/TACE/operation/others)	-	30/9/14/1
Treatment (curative/non-curative)	-	54/0
Duration from the last HCC treatment to the initiation of DAA therapy (months) ^a^	-	5.9 (1–95.2)

Abbreviations: HCV, hepatitis C virus; AST, aspartate aminotransferase; ALT, alanine aminotransferase; FIB-4, fibrosis 4; M2BPGi, Mac-2 binding protein glycosylation isomer; AFP, alpha-fetoprotein; HCC, hepatocellular carcinoma; RFA, radiofrequency ablation; TACE, transcatheter arterial chemoembolization. ^a^ Data are shown as median values (range). ^b^ Observation period for patients without HCC development after DAA treatment.

**Table 2:** Baseline factors associated with HCC occurrence after DAA therapy.

	Cut-off Value	Univariate *p*-Values	Multivariate		
Factors			HR	95% CI	*p*-Values
Age (years)	≥75	* 0.026	2.92	1.34–6.33	* 0.007
Sex (male/female)	Male	0.459			
HCV genotype	1	0.436			
Platelet count (10^4^/μL)	≥13.8	0.5			
Albumin (g/dL)	≥4.0	0.393			
AST (IU/L)	≥47	0.079			
ALT (IU/L)	≥42	0.829			
FIB-4 index	≥4.55	0.093			
M2BPGi	≥4.23	* <0.001	2.13	0.82–5.52	0.12
AFP (ng/mL)	≥7.7	* 0.006	1.61	0.66–3.89	0.292
Angiopoietin-2 (pg/mL)	≥464	* 0.003	2.75	1.18–6.37	* 0.019
Delta Angiopoietin-2 between baseline and end of treatment	≥−13	0.258			
Diabetes, *n* (%)	Yes	* 0.03	2.05	0.89–4.69	0.091

Abbreviations: HCV, hepatitis C virus; AST, aspartate aminotransferase; ALT, alanine aminotransferase; FIB-4, fibrosis 4; M2BPGi, Mac-2 binding protein glycosylation isomer; AFP, alpha-fetoprotein; HCC, hepatocellular carcinoma. * Statistically significant difference, *p* < 0.05.

**Table 3:** Factors at the end of treatment associated with HCC occurrence after DAA therapy.

	Cut-off Value	Univariate *p*-Values	Multivariate		
Factors			HR	95% CI	*p*-Values
Age (years)	≥75	* 0.026	1.85	0.8–4.29	0.151
Sex (male/female)	Male	0.459			
HCV genotype	1	0.436			
Platelet count (10^4^/μL) at EOT	≥15.8	0.765			
Albumin (g/dL) at EOT	≥4.3	0.404			
AST (IU/L) at EOT	≥24	0.194			
ALT (IU/L) at EOT	≥18	0.757			
FIB-4 index at EOT	≥3.67	* 0.046	1.42	0.57–3.51	0.448
M2BPGi at EOT	≥1.89	* 0.001	1.37	0.5–3.79	0.542
AFP (ng/mL) at EOT	≥4.6	* 0.037	1.38	0.57–3.34	0.48
Angiopoietin-2 (pg/mL) at EOT	≥402	* <0.001	3.68	1.37–9.9	* 0.01
Diabetes, *n* (%)	Yes	* 0.03	2.05	0.85–4.93	0.109

Abbreviations: HCV, hepatitis C virus; EOT, end of treatment; AST, aspartate aminotransferase; ALT, alanine aminotransferase; FIB-4, fibrosis 4; M2BPGi, Mac-2 binding protein glycosylation isomer; AFP, alpha-fetoprotein; HCC, hepatocellular carcinoma. * Statistically significant difference, *p* < 0.05.

**Table 4:** Baseline factors associated with recurrent HCC after DAA therapy.

	Cut-off Value	Univariate *p*-Values	Multivariate		
Factors			HR	95% CI	*p*-Values
Age (years)	≥71	0.605			
Sex (male/female)	Male	0.407			
HCV genotype	1	0.255			
Platelet count (10^4^/μL)	≥13.4	0.936			
Albumin (g/dL)	≥3.7	0.517			
AST (IU/L)	≥45	* <0.001	1.81	0.67–4.93	0.244
ALT (IU/L)	≥37	* <0.001	2.61	0.9–7.58	0.077
FIB-4 index	≥6.4	0.872			
M2BPGi	≥5.22	* 0.025	1.332	0.751–1.492	0.213
AFP (ng/mL)	≥8.6	0.053			
Angiopoietin-2 (pg/mL)	≥446	* 0.017	2.659	1.209–5.847	* 0.015
Delta Angiopoietin-2 (pg/mL) between baseline and end of treatment	≥−31	0.197			
Diabetes, *n* (%)	Yes	0.409			
Previous HCC characteristics					
HCC stage	>1	0.509			
Treatment	RFA or operation	0.685			
Duration from HCC treatment to DAA (months)	≥7.62	0.494			

Abbreviations: HCV, hepatitis C virus; AST, aspartate aminotransferase; ALT, alanine aminotransferase; FIB-4, fibrosis 4; M2BPGi, Mac-2 binding protein glycosylation isomer; AFP, alpha-fetoprotein; HCC, hepatocellular carcinoma; RFA, radiofrequency ablation; TACE, transcatheter arterial chemoembolization. * Statistically significant difference, *p* < 0.05.

## Data Availability

All relevant data within the manuscript and its Supplementary Materials.

## Supplementary Materials

The following supporting information can be downloaded at: https://www.mdpi.com/article/10.3390/v15010181/s1, Table S1: Baseline patient characteristics.
