# Supplementary material for: Serum Angiopoietin-2 Predicts the Occurrence and Recurrence of Hepatocellular Carcinoma after Direct-Acting Antiviral Therapy for Hepatitis C
Source: Viruses. 2023 Jan 7;15(1):181. doi: 10.3390/v15010181 (PMC9862289; doi:10.3390/v15010181)
Supplement: Supplementary file 1 [file viruses-15-00181-s001.zip › viruses-2122167-supplementary.pdf]

Supplementary Table S1 Baseline patient characteristics

Number	310
Age (years)[a]	71 (22–86)
Sex (male/female)	131/179
HCV-genotype (1/2/unknown)	232/76/2
Platelet count (10^4/μL) [a]	13 (1.9–38.8)
Albumin (g/dL) [a]	3.9 (2.5-4.9)
AST (IU/L) [a]	47 (15–342)
ALT (IU/L) [a]	41 (6–379)
FIB-4 index [a]	4.21 (0.54–39.35)
M2BPGI [a]	2.86 (0.28–20.01)
AFP (ng/mL) [a]	5.8 (1.1–250.8)
Angiopoietin-2 (pg/mL) [a]	525 (207–1599)
Diabetes, n (%)	64 (21%)
Duration from start-date of DAA treatment (months) [a,b]	50.6 (12.1–86)
History of HCC, n (%)	54 (17%)
**Previous HCC characteristics**	
HCC Stage (1/2/3/4)	30/23/1/0
Treatment (RFA/TACE/Operation/Other)	30/9/14/1
Treatment (curative/non-curative)	54/0
Duration from last HCC treatment to DAA initiation (months) [a]	5.9 (1–95.2)
**Development of HCC after DAA therapy, n (%)**	66 (21%)
Duration from the start-date of DAA treatment to HCC development (months) [a]	22.8 (6.2–66)

Abbreviations: HCV, Hepatitis C virus; AST, aspartate aminotransferase; ALT, alanine aminotransferase; FIB-4, fibrosis 4; M2BPGI, Mac-2 binding protein glycosylation isomer; AFP, alpha-fetoprotein; HCC, hepatocellular carcinoma; RFA, radiofrequency ablation; TACE, transcatheter arterial chemoembolization. [a]Data are shown as median values (range). [b]Observation period for patients without HCC development after DAA treatment.
